# KIF2A Overexpression and Its Association with Clinicopathologic Characteristics and Poor Prognoses in Patients with Gastric Cancer

**DOI:** 10.1155/2016/7484516

**Published:** 2016-09-28

**Authors:** Shu Zhang, Fang Huang, Yan Wang, Qinjie Song, Xiaobing Yang, Han Wu

**Affiliations:** ^1^Department of Pathology, Nantong University Affiliated Hospital, Nantong, Jiangsu 226001, China; ^2^Department of General Surgery, Nantong University Affiliated Hospital, Nantong, Jiangsu 226001, China

## Abstract

Kinesin family protein 2A (KIF2A), an M-type nonmotile microtubule depolymerase, has attracted attention for its role in carcinogenesis and poor prognoses in various human cancers. In this study, we aimed to evaluate the expression of KIF2A and its robustness and potential to predict clinical outcomes in gastric cancer (GC) patients. The messenger RNA (mRNA) expression of KIF2A was determined in 24 pairs of cancerous and adjacent nontumor tissues by real-time polymerase chain reaction. Immunohistochemistry of KIF2A was performed on a tissue microarray composed of 461 GC and 65 matched adjacent nontumor tissues removed during surgeries and 18 chronic gastritis, 15 intestinal metaplasia, and 37 low-grade and 62 high-grade intraepithelial neoplasias acquired through gastric endoscopic biopsies. Univariate and multivariate Cox regression models were used to perform survival analyses. The high KIF2A expression was significantly correlated to histological type, TNM stage, and lymph node metastasis. A negative correlation was found between KIF2A expression and the 5-year survival rate of GC patients. In addition, multivariate analysis indicated that KIF2A is an independent prognostic factor in GC. This study demonstrated the high KIF2A expression might serve as an independent marker for poor prognoses in GC patients.

## 1. Introduction

Gastric cancer (GC) is one of the most prevalent and aggressive cancers worldwide with 952,000 new cases diagnosed annually [1, 2]. Approximately 70% of GC cases seen are from developing countries. In China, GC is the third leading cause of death from all cancers, with an age-standardized incidence of 22.7/100,000 [3]. Notwithstanding rapid advances in treatments, including surgery, chemotherapy, and targeted therapy, the prognosis for GC patients is far from satisfying [4]. High rates of metastasis and recurrence are major obstacles to improving long-term survival after a curative resection [5, 6]; therefore, new molecular prognostic markers and therapeutic targets are needed to improve the clinical outcome for patients with this disease. Although countless efforts have been made to pinpoint reliable GC prognostic biomarkers based on tumor biology [7], many issues, such as reproducibility and specificity, still need to be addressed and much work is needed to identify high-quality GC prognostic markers.

Studies have shown that cytoskeletal reorganizations play an important role in the migration of neoplastic cells [8]. Microtubules (MTs), the fundamental component of the cytoskeleton, are essential not only for mitotic activity of malignant cells but also for invading neighboring tissues and causing distant metastasis [9]. The decrease and depolymerization of MTs are associated with the metastatic potential of the malignant tumors. The kinesin-13 family members, including kinesin superfamily protein 2A (KIF2A) and KIF2B, and the mitotic centromere-associated kinesin (MCAK), are M-type nonmotile microtubule depolymerases and play central role in regulating microtubule dynamics during mitotic progression [10, 11]. The spindle, a microtubule-based structure, is required for accurate chromosome segregation in both the mitotic and meiotic cell cycles [9]. KIF2A is a microtubule minus an end-depolymerizing motor and is essential in assembling normal bipolar spindles and mitosis progression [12]. With the depletion of KIF2A, cells form monopolar spindles, which might result in chromosome gain or loss in the human pancreatic cancer line CFPAC-1 and osteosarcoma cell line U2OS [10, 13]. As a result, cancer cell-cycle progression is also halted [10, 13]. Interestingly, several lines of evidence have indicated that KIF2A might be implicated in carcinogenesis and the development of drug resistance in cancer cells [14]. The abnormal expression and dysfunction of KIF2A are associated with tumorigenesis and the progression of certain types of human cancers [15]. Silencing the KIF2A gene suppressed the proliferation of squamous cell carcinoma of the tongue (SCCT, cell line Tca8113) and synergized the tumor suppression effect of 5-fluorouracil in a nude-mice model [16]. Furthermore, it was demonstrated that patients with a high expression of KIF2A tend to have a poor prognosis for certain types of human cancers. For example, upregulation of KIF2A is associated with the metastasis and poor prognosis of colorectal cancer [17], breast cancer [18], and laryngeal squamous cell carcinoma [19]; however, until now, very few studies on the relationship between KIF2A and other malignant tumors have been reported and the expression of KIF2A and its prognostic role in GC have not yet been explored. With this in mind, we examined the expression of KIF2A in GC and evaluated its association with the disease's progression, invasion, and metastasis.

## 2. Materials and Methods

### 2.1. Subject Characteristics

Six hundred fifty-eight formalin-fixed, paraffin-embedded tissue samples from patients with GC (*n* = 461) and paired adjacent nontumor tissues (*n* = 65) taken during surgical procedures and chronic gastritis (*n* = 18), intestinal metaplasia (*n* = 15), low-grade intraepithelial neoplasia (*n* = 37), and high-grade intraepithelial neoplasia (*n* = 62) acquired through gastric endoscopic biopsies were used in this study. All tissue blocks were randomly obtained from the clinical biobank of Affiliated Hospital of Nantong University between January 2005 and December 2010.

In terms of the 461 GC patients, none of them had received any type of treatment before surgery. All samples were histopathologically confirmed by at least two independent pathologists. Median cancer patient age was 55.2 years (range: 27.5 and 78.4 years). Clinical characteristics as well as follow-up data were retrieved retrospectively from patients' medical records and clinical biobank of Affiliated Hospital of Nantong University. The 1-, 3-, and 5-year overall survival (OS) of the cohort were 80.52, 61.36, and 42.27%. At last follow-up, 211 (45.82%) patients had died from either recurrence of the disease (*n* = 177) or surgery-related complications without recurrence (*n* = 34). Among the remaining 250 patients, the mean duration of follow-up was 43.7 months (range: 16.4–59.6 months, standard deviation: ±9.2). OS was defined as the interval between the initial biopsy-confirmed diagnosis and death or between the initial biopsy-confirmed diagnosis and the last follow-up for surviving patients.

The study protocol was approved by the Human Research Ethics Committee of Nantong University Affiliated Hospital. All of the procedures were done in accordance with the Declaration of Helsinki and relevant policies in China.

### 2.2. Tissue Microarray Construction and Immunohistochemistry

Tissue microarrays (TMAs) were constructed as previously described [3]. In brief, TMAs were generated using the UT06 Quick-Ray Manual Tissue Microarrayer (Unitma Co., Ltd., Seoul, South Korea). Immunohistochemistry (IHC) analysis was performed according to standard protocols and carried out using a mouse monoclonal anti-human KIF2A antibody (dilution 1 : 100) (Abcam, Cambridge, MA, USA). Reactions were detected using the Envision^+^ *™* peroxidase kit (Dako, Carpinteria, CA, USA). Samples were incubated with 3,3′-diaminobenzidine plus (Dako, Carpinteria, CA, USA), then counterstained with hematoxylin, dehydrated with graded alcohol, cleared in xylene, and placed in permanent mounting media with a cover slip.

All cases were reviewed and scored by researchers blinded to the clinical characteristics of the patients. KIF2A expression was scored using the semiquantitative H-score method, taking into account both the staining intensity and the percentage of cells that stained at that intensity. The staining intensity was scored as 0 (no stain), 1+ (weak stain), 2+ (moderate stain), or 3+ (intense stain). The percentage of cells that stained at each intensity was determined and multiplied by the intensity score to produce an intensity percentage score. The scores representing the percentage of positive cells were as follows: 0, 0–20%; 1, 21–50%, 2, 51–75%; 3, 76–100%. The final staining scores were calculated by adding the four intensity percentage scores. The staining score had a minimum value of 0 (no stain) and a maximum value of 300 (100% of cells with a 3+ staining intensity).

### 2.3. Quantitative Real-Time Polymerase Chain Reaction

Fresh frozen tumor tissue samples (*n* = 24) and matched adjacent samples were collected for quantitative real-time polymerase chain reaction (qRT-PCR) analysis to investigate the difference of KIF2A expression between cancerous and normal tissues. Total tissue ribonucleic acid (RNA) was extracted using the RNeasy Mini Kit (Qiagen, Valencia, CA, USA). qRT-PCR analysis was performed according to the manufacturer's instructions (Quant SYBR Green PCR Kit, Tiangen Biotech, Beijing, China). The KIF2A primer sequences were as follows: forward 5′-GCCTTTGATGACTCAGCTCC-3′ and reverse 5′-TTCCTGAAAAGTCACCACCC-3′. Moreover, the glyceraldehyde-3-phosphate dehydrogenase (GAPDH) gene as the housekeeper gene was run in parallel to normalize KIF2A gene expression (forward primer 5′-TGCACCACCAACTGCTTAGC-3′ and reverse primer 3′-GGCATGGACTGTGGTCATGAG-5′). For relative quantification, 2^−ΔΔCt^ was calculated and used as an indicator of the level of enzyme expression. All analyses of the samples were performed in triplicate.

### 2.4. Statistical Analysis

The statistical analyses were performed using SPSS 22.0 (IBM Corporation, Armonk, NY, USA). Cutoff values for high or low KIF2A expression were determined using X-tile (Rimm Lab at Yale University, http://www.tissuearray.org/rimmlab) [20]. The relationship between KIF2A expression and clinicopathologic variables was estimated using the *χ* ^2^ test. Survival curves were analyzed using the Kaplan-Meier method and compared by log-rank test. The Cox-regression model was used for univariate and multivariate analyses, in which all of the clinicopathologic features served as covariates. Statistical significance was established at *p* < 0.05 (two-tailed).

## 3. Results

### 3.1. KIF2A Expression in Gastric Cancer Tissues

**Figure 1:**
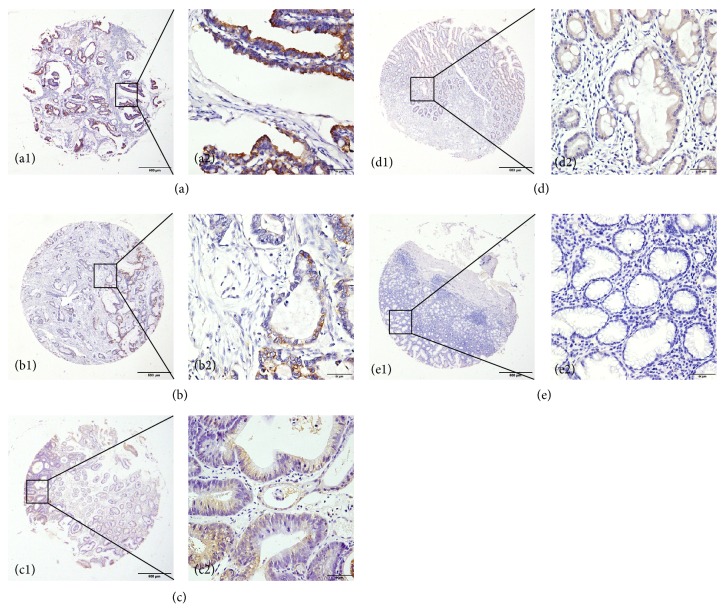
KIF2A expression in benign and malignant gastric tissue samples in TMA sections. (a) Gastric cancer tissue with strongly positive KIF2A staining; (b) high-grade intraepithelial neoplasia with moderately positive KIF2A staining; (c) low-grade intraepithelial neoplasia with moderately positive KIF2A staining; (d) intestinal metaplasia with weakly positive KIF2A staining; (e) chronic gastritis with KIF2A-negative staining. Columns 1 and 3 and 2 and 4 are KIF2A staining at a magnification of ×40 (bar = 500 *μ*m) and ×400 (bar = 50 *μ*m), respectively.

We explored KIF2A expression by performing IHC analysis on TMAs comprising 461 GC and 65 matched tumor adjacent tissues from patients with GC. We also examined KIF2A expression in 18 chronic gastritis, 15 intestinal metaplasia, and 37 low-grade and 62 high-grade intraepithelial neoplasias. IHC analysis of TMA sections showed that KIF2A was expressed mainly in the tumor epithelial cells, and staining showed that it occurred primarily in the cytoplasm (Figure 1). Overall, only a small proportion of chronic gastritis (27.78%, 5/18) and tumor adjacent tissues (29.23%, 19/65) displayed high KIF2A expression, whereas high expression of this protein was detected in 43.24% (16/37) of low-grade intraepithelial neoplasia, 59.68% (37/62) of high-grade intraepithelial neoplasia, 73.33% (11/15) of intestinal metaplasia, and 64.86% (299/461) of GCs (*p* < 0.001) (Table 1).

**Figure 2:**
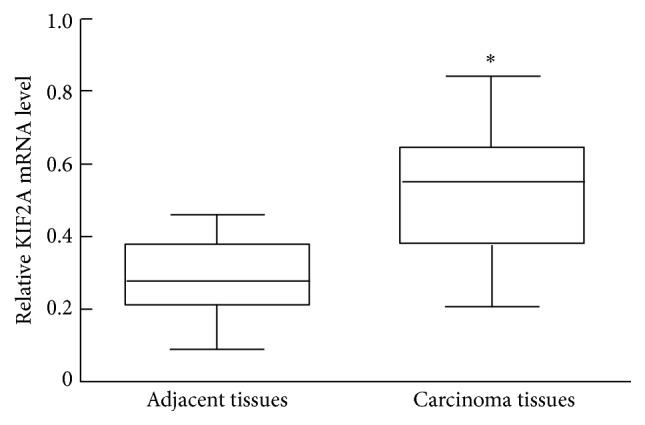
KIF2A mRNA levels in gastric cancer and paired adjacent tissues determined by real-time polymerase chain reaction. KIF2A expression was significantly higher in the cancerous tissue than in their paired peritumoral counterparts (^*∗*^ *p* < 0.0001).

To determine whether KIF2A mRNA expression differs between GC and normal tissues, we also analyzed KIF2A gene levels in tissue samples from 24 patients with GC and compared them with the corresponding peritumoral tissue samples. KIF2A expression was significantly higher in the cancerous tissue samples than in their paired peritumoral counterparts (*p* < 0.001) (Figure 2).

### 3.2. Relationship between KIF2A Expression and Clinicopathologic Characteristics

To determine whether KIF2A is important in determining the clinical outcomes for patients with GC, we examined the relationship between the KIF2A expression and clinical parameters of these patients. High expression of KIF2A correlated significantly with the patient's age (*p* = 0.021), tumor histology (*p* = 0.039), TNM stage (*p* = 0.008), and lymph node metastasis (*p* = 0.009). The proportion of tumors with high KIF2A expression increased with the degree of lymph node metastasis (N0, 56.25%; N1, 65.17%; N2, 69.15%; and N3, 75.49%) (Table 2). These results suggested that GCs with high cytoplasmic KIF2A expression are prone to progress to a more advanced stage and lymph node metastasis than those with low KIF2A expression.

### 3.3. Prognostic Value of KIF2A Expression in Gastric Cancer

**Figure 3:**
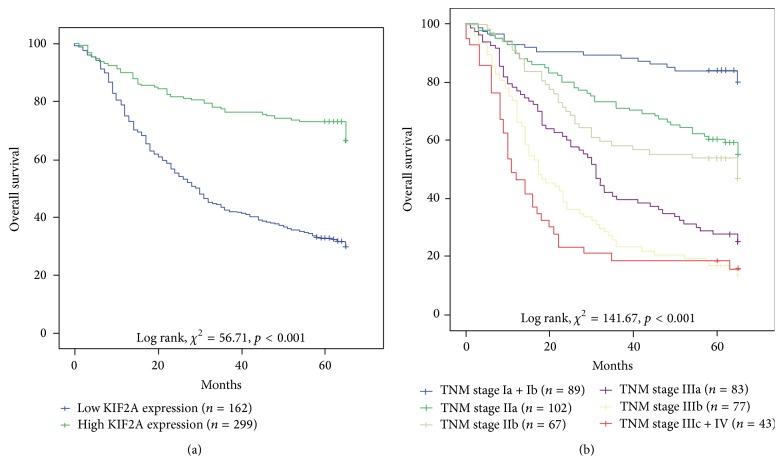
Kaplan-Meier survival curves for gastric cancer, with comparisons evaluated using the log-rank test. (a) Overall survival curves of a high rate of expression of kinesin family protein 2A (KIF2A) and a low rate of expression of KIF2A; (b) overall survival curves by tumor status, lymph node metastasis, and distant metastasis (TNM) stage.

The X-tile-based TMA data analysis indicated a significant cutoff point for OS of GC patients. For KIF2A, the cutoff selected was 130 (i.e., a score of from 0 to 130 represented a low rate of expression [KIF2A-low] and a score of from 131 to 300 represented a high rate of expression [KIF2A-high]). The univariate analysis revealed that KIF2A-high correlated significantly with a poor OS (hazard ratio [HR]: 0.334; 95% confidence interval [CI]: 0.246–0.453; *p* < 0.001) and with previously reported prognostic markers, including differentiation (HR: 1.38; 95% CI: 1.146–1.661; *p* = 0.001), TNM stage (HR: 1.563; 95% CI: 1.444–1.691; *p* < 0.001), lymph node metastasis (HR: 1.648; 95% CI: 1.483–1.833; *p* < 0.001), and distant metastasis (HR: 3.167; 95% CI: 2.518–4.646; *p* < 0.001) (Table 3). Individual clinicopathological features that demonstrated significance in the univariate analysis were adopted as covariates in a multivariate Cox proportional hazards model and were further analyzed. Multivariate Cox proportional hazards analysis also revealed that KIF2A was an independent prognostic marker associated with OS (HR: 0.366; 95% CI: 0.268–0.5; *p* < 0.001), as well as with tumor differentiation (HR: 1.229; 95% CI: 1.004–1.505; *p* = 0.046) and advanced tumor stage (HR: 1.324; 95% CI: 1.113–1.575; *p* = 0.002) (Table 3). Kaplan-Meier survival analysis showed that GC with KIF2A-high had a significantly worse prognosis than that with KIF2A-low (*p* < 0.001) (Figure 3). Likewise, tumor TNM stage also influenced the survival of patients with early TNM stage predicting a favorable prognosis.

## 4. Discussion

In this study, we found that KIF2A mRNA and protein levels in GC tissue were highly expressed compared to that in adjacent normal tissue. We further confirmed the increased prevalence of high KIF2A expression in cancerous tissues (low- and high-grade intraepithelial neoplasia) as compared to noncancerous tissues (chronic gastritis). The increase in KIF2A expression was associated with a decrease in patient survival time, indicating that KIF2A is a potential new biomarker in the prognosis for GC patients. We also demonstrated that patients with lymph node metastasis had a high frequency of KIF2A overexpression.

MTs, as the vital components of the cytoskeleton, play important roles in mitosis, cell migration, cell signaling, and trafficking. As a member of the kinesin-13 family, KIF2A is responsible for the assembly of bipolar spindles, which are crucial for normal mitosis and chromosome segregation of cells [21]. According to recent studies, KIF2A is present throughout the cell and might accelerate MT turnover, resulting in an increase in cancer cell motility [8, 22]. This result might partially explain the finding of higher rates of lymphatic invasion and metastasis and a poorer prognosis in cancer patients with high levels of KIF2A. Moreover, in our results, clinicopathological analysis revealed that GC with high KIF2A expression was associated with histological type, advanced TNM stage, and lymph node metastasis. Although few investigations have explored the role of KIF2A in cancers, there are some published findings in accordance with our results. Studies found that knockdown of KIF2A suppressed the proliferation, migration, and invasion of breast cancer cell line MDA-MB-231 cells and SCCT cell line Tca8113 cells [18, 23]. And KIF2A expression in breast cancer tissue with lymph node metastasis and HER2 positive cancer was higher than that in cancer tissue without. Similarly, a high expression of KIF2A was more frequently detected in SCCT than in the corresponding adjacent tissues and was correlated with the progressive phenotype of the disease [16, 23]. KIF2A expression in colorectal cancer exceeded that in normal adjacent tissues and negatively correlated with OS of colorectal cancer patients [17]. It was recently demonstrated that expression levels of KIF2A were significantly higher in grades III-IV glioma tissues compared with those in grades I-II glioma tissues [24].

Unfortunately, the underlying mechanism by which overexpression of KIF2A contributes to cancer progression remains unclear; therefore the identification of KIF2A's regulation of the MT network of cells, especially tumor cells, could lead to a better understanding of the regulation of tumor progression and will be helpful in improving cancer therapy. It was recently determined that KIF2A and MCAK were upregulated in KRAS-dependent transformed human bronchial epithelial cells (HBECs). Knocking down either KIF2A or MCAK reduced the ability of KRAS^G12V^-expressing transformed HBECs to migrate and invade, suggesting that aberrant expression of these proteins during transformation can contribute to the migratory potential of cancer cells [25, 26]. These findings suggest that targeting the Ras-mediated pathways that promote different aspects of cancer biology could be a therapeutic advantage. In addition, the extracellular signal-regulated kinase (ERK)1/2 pathway was presumed as a major Ras effector that controls the expression of these kinesins [25, 26]. The Raf/ERK1/2 and phosphatidylinositol-3-kinase (PI3K) pathways are major effectors of Ras transformation and have powerful actions on the cytoskeleton [27, 28]. Interestingly, it was recently reported that silencing siRNA-mediated KIF2A inhibited the PI3K/protein kinase B (AKT) pathway in Tca8113 cells and led to cell apoptosis [29]. Once stimulated, PI3K sequentially activates AKT as well as downstream signaling cascades to regulate multiple important cellular events, such as proliferation, survival, apoptosis, and migration [30, 31]. Excessive activity of the PI3K/AKT signaling pathway has been implicated in the carcinogenesis of a variety of human cancers [32, 33]. Collectively, these findings suggest that KIF2A might promote tumor growth and invasion partially through stimulating the PI3K/AKT signaling pathway. And as an end-binding protein (EB)1/3-binding kinase, tau-tubulin kinase 2 with EB1/3 phosphorylated KIF2A and antagonized KIF2A-induced depolymerization at MTs plus ends for cell migration [34].

In spite of our findings, limitations of our study need to be addressed. We lacked* in vitro* data to verify our results and although we did examine the function of KIF2A in the GC cell lines, further studies are necessary to investigate the underlying mechanisms by which KIF2A influences the invasion and metastasis of cancer cells.

## 5. Conclusions

The expression of KIF2A in GC is upregulated when compared to that in normal gastric tissue, and this overexpression is associated with lymph node metastasis, advanced TNM stages, and a poor prognosis; therefore, we suggest that KIF2A could be a prognostic marker, even a molecular target, in GC therapy.

## Figures and Tables

**Table 1:** KIF2A expression in gastric benign and malignant tissues.

Characteristic	*n*	KIF2A− (%)	KIF2A+ (%)	Pearson *χ* ^2^	*p*
Total	658	271 (41.19)	387 (58.81)	42.4355	<0.001^*∗*^
Chronic gastritis	18	13 (72.22)	5 (27.78)		
Intestinal metaplasia	15	4 (26.67)	11 (73.33)		
Low-grade intraepithelial neoplasia	37	21 (56.76)	16 (43.24)		
High-grade intraepithelial neoplasia	62	25 (40.32)	37 (59.68)		
Cancer	461	162 (35.14)	299 (64.86)		
Surgical margin	65	46 (70.77)	19 (29.23)		

^*∗*^ *p* < 0.05; KIF2A+ represents high KIF2A expression; KIF2A− represents low KIF2A expression.

**Table 2:** Association of high expression of KIF2A with clinicopathological characteristics in gastric cancer patients.

Characteristic	*n*	KIF2A−	KIF2A+	Pearson *χ* ^2^	*p*
Total	461				
Gender				1.1992	0.273
Male	344	116 (33.72)	228 (66.28)		
Female	117	46 (39.32)	71 (60.28)		
Age				5.3135	0.021^*∗*^
<60	156	66 (42.31)	90 (57.69)		
≧60	305	96 (31.48)	209 (68.52)		
Histological type				10.0769	0.039^*∗*^
Tubular	384	128 (33.38)	256 (66.67)		
Mixed (tubular and mucinous)	17	6 (35.29)	11 (64.71)		
Mucinous	25	10 (40.00)	15 (60.00)		
Signet ring cell	15	11 (73.33)	4 (26.67)		
Others^a^	20	8 (40.00)	12 (60.00)		
Differentiation				1.8888	0.169
Well and middle	143	43 (30.07)	100 (69.93)		
Poor	280	103 (36.79)	177 (63.21)		
Others^b^	38	16	22		
TNM stage				17.5014	0.008^*∗*^
Ia	37	21 (56.76)	16 (43.24)		
Ib	52	21 (40.38)	31 (59.62)		
IIa	102	43 (42.16)	59 (57.84)		
IIb	67	22 (32.84)	45 (67.16)		
IIIa	83	26 (31.33)	57 (68.67)		
IIIb	77	18 (23.38)	59 (76.62)		
IIIc + IV	43	11 (25.58)	32 (74.42)		
T				5.7839	0.123
Tis	9	6 (66.67)	3 (33.33)		
T1	41	20 (48.78)	21 (51.22)		
T2	94	32 (34.04)	62 (65.96)		
T3 + T4	317	117 (36.91)	200 (63.09)		
N				11.5439	0.009^*∗*^
N0	176	77 (43.75)	99 (56.25)		
N1	89	31 (34.83)	58 (65.17)		
N2	94	29 (30.85)	65 (69.15)		
N3	102	25 (24.51)	77 (75.49)		
M				2.5075	0.113
M0	426	154 (36.15)	272 (63.85)		
M1	35	8 (22.86)	27 (77.14)		
Preoperative CEA, ng/mL				2.6563	0.103
≦5	185	73 (39.46)	112 (60.54)		
>5	61	17 (27.87)	44 (72.13)		
Unknown	215	72	143		
Preoperative CA199, U/mL				0.1195	0.730
≦37	200	74 (37.00)	126 (63.00)		
>37	41	14 (34.15)	27 (65.85)		
Unknown	220	74	146		

^*∗*^ *p* < 0.05; ^a^others: papillary adenocarcinoma, 3 cases; adenosquamous carcinoma, 3 cases; squamous cell carcinoma, 3 cases; undifferentiated carcinoma, 1 case; small cell malignant tumor, 8 cases; carcinoid, 1 case; focal cancer, 1 case.

^b^others: besides tubular and papillary adenocarcinoma.

**Table 3:** Univariate and multivariate analysis of prognostic factors for overall survival in gastric cancer patients.

	Univariate analysis				Multivariate analysis			
	HR	*p* > |*z*|	95% CI		HR	*p* > |*z*|	95% CI	
KIF2A expression								
High versus low	0.334	<0.001^*∗*^	0.246	0.453	0.366	<0.001^*∗*^	0.268	0.500
Age (years)								
≤60 versus >60	1.402	0.012^*∗*^	1.077	1.827	1.231	0.140	0.934	1.622
Gender								
Male versus female	1.042	0.777	0.786	1.381				
Histological type								
Tubular versus mixed (tubular and mucinous) versus Mucinous versus	1.015	0.860	0.856	1.205				
signet ring cell carcinoma versus others^a^								
Differentiation								
Well versus middle versus poor	1.380	0.001^*∗*^	1.146	1.661	1.229	0.046^*∗*^	1.004	1.505
TNM stage								
0 versus Ia versus Ib versus IIa versus IIb versus IIIa versus IIIb versus IIIc and versus IV	1.563	<0.001^*∗*^	1.444	1.691	1.324	0.002^*∗*^	1.113	1.575
T								
Tis versus T1 versus T2 versus T3 + T4	1.079	0.113	0.982	1.185				
N								
N0 versus N1 versus N2 versus N3	1.648	<0.001^*∗*^	1.483	1.833	1.183	0.096	0.971	1.441
M								
M0 versus M1	3.167	<0.001^*∗*^	2.518	4.646	1.741	0.102	0.896	3.383
Preoperative CEA (ng/mL)								
≤5 versus ≥5	1.136	0.053	0.998	1.293				
Preoperative CA199 (U/mL)								
≤37 versus >37	1.102	0.129	0.972	1.251				

^*∗*^ *p* < 0.05; ^a^others: papillary adenocarcinoma, 3 cases; adenosquamous carcinoma, 3 cases; squamous cell carcinoma, 3 cases; undifferentiated carcinoma, 1 case; small cell malignant tumor, 8 cases; carcinoid, 1 case; focal cancer, 1 case.
